# Novel Polyelectrolytes Based on Naphthalene Diimide with Different Counteranions for Cathode Interlayers in Polymer Solar Cells

**DOI:** 10.3390/ijms25010522

**Published:** 2023-12-30

**Authors:** Rahmatia Fitri Binti Nasrun, Dong Hwan Son, Joo Hyun Kim

**Affiliations:** 1 Department of Polymer Engineering, Pukyong National University, Busan 48513, Republic of Korea; rahmatiaf@pukyong.ac.kr (R.F.B.N.); ehdghks507@naver.com (D.H.S.); 2 CECS Research Institute, Core Research Institute, Busan 48513, Republic of Korea

**Keywords:** polyelectrolytes, cathode interlayer, naphthalene diimide, polymer solar cell

## Abstract

We synthesized novel polyelectrolytes based on naphthalene diimide with quaternary amine featuring hydroxyl groups at the side chain, along with different counteranions (PF-NDIN-Br-OH and PF-NDIN-I-OH) for polymer solar cell (PSC) application as the interlayer. The polyelectrolytes establish a beneficial interface dipole through the ionic moieties and synergistic effects arising from the hydroxyl groups located at the side chain. Incorporating polyelectrolytes as the cathode interlayer resulted in an enhancement of the power conversion efficiency (PCE). The PCE of the device with PF-NDIN-Br-OH increased from 8.96% to 9.51% compared to the ZnO-only device. The best PCE was obtained with the device based on PF-NDIN-I-OH, up to 9.59% resulting from the $J_{sc}$ enhancement. This outcome implies a correlation between the performance of the device and the synergistic effects observed in polyelectrolytes containing hydroxyl groups in the side chain, along with larger anions when employed in PSCs.

## 1. Introduction

Polymer solar cells (PSCs) are increasingly acknowledged as a potential renewable energy technology, owing to their light weight, cost-effectiveness, and flexibility [1,2,3,4,5,6,7]. As a result, the power conversion efficiency (PCE) of PSCs has increased by more than 20% [8,9,10,11,12,13,14,15]. These advancements were accomplished by designing suitable active layers to enhance morphology and optimize interfacial properties [16]. Recognizing the crucial role of interlayers in attaining high-performance PSCs, numerous studies have been conducted to improve interfacial properties by integrating various interlayers [17,18]. Interlayers play an important role in enhancing exciton collection by reducing the cathode interface barrier and inhibiting charge recombination. Consequently, the improvement in short-circuit current density ($J_{sc}$), open-circuit voltage ($V_{oc}$), and fill factor (FF) in a device will occur [19,20].

A cathode interlayer positioned between the photoactive layer and the cathode serves the dual function of electron extraction and hole blocking. Ideal materials for cathode interlayers should possess the capability to lower the work function (WF) of electrodes, ensuring alignment of energy levels with photoactive layers. Additionally, ensuring high conductivity and electron mobility is essential to support the use of thick cathode interlayers, a critical factor in the roll-to-roll manufacturing process of PSCs. The research focus has shifted toward organic cathode interlayer materials due to their intrinsic qualities, including flexibility and strong compatibility with the photoactive layer. Incorporating versatile organic groups is easily achievable, enhancing charge transport with the photoactive layer. Conjugated electrolytes that incorporate ionic species within either the polymer main chain or side chains have proven to be the most successful organic cathode interlayers to enhance PCE [21]. Polymer chains exhibit inherent structural properties, and the morphology can influence optical and electrochemical characteristics [22]. Polyelectrolytes are commonly synthesized by integrating an ionic moiety into the side chain of the polymer, resulting in excellent solubility in alcohol. This method can be implemented by employing quaternizing reagents such as bromoethanol and iodoethanol to generate the hydrophilic quaternary ammonium salt [23,24]. 

**Figure 1:**
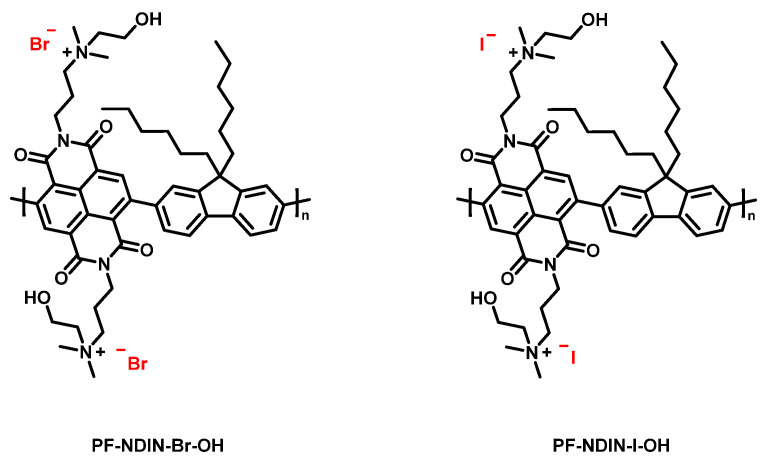
Chemical structure of polymers.

**Scheme 1:**
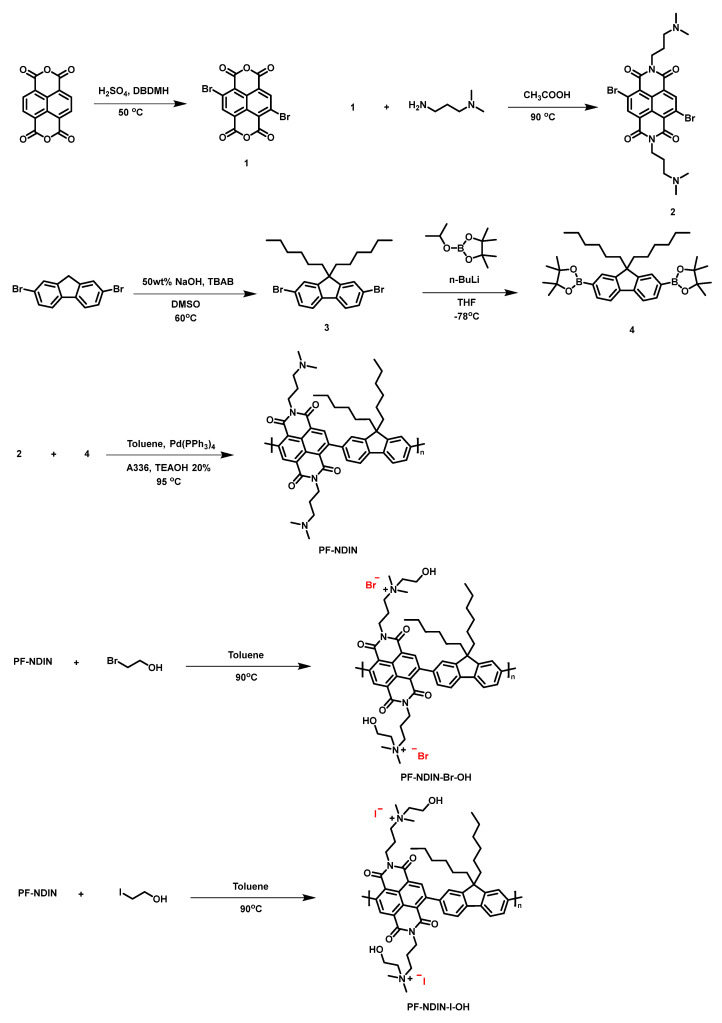
Synthesis route of CPEs.

The ionic charges offer polyelectrolytes a straightforward process for adjusting interaction between molecules and fine-tuning the optical properties [25]. Additionally, the interface properties can be finely tuned by different types of counteranions (CAs). Research has demonstrated that the WF of electrodes and ZnO can be modified by varying the counteranion [5,26,27,28]. Polyelectrolytes with various CAs have been utilized to maximize the collection of charge capability at the cathode interface [29]. Furthermore, previous studies have shown that the modification of polyelectrolytes with side chains containing hydroxyl groups can impact the PCE [30]. In the present study, we aim to integrate both of these approaches to examine the synergistic effects of polyelectrolytes with hydroxyl groups at the side chain and larger anions (e.g., iodide) when applied to PSCs [31,32,33,34]. We synthesized novel polyelectrolytes based on naphthalene diimide with quaternary ammonium salt featuring hydroxyl groups at the end of the side chain, along with different counteranions (Figure 1 and Scheme 1) [35]. The devices achieved a high PCE of up to 9.59%, attributed to the generation of a beneficial interface dipole. This study could serve as an initial exploration for enhancing the performance by simply changing the anion and demonstrated the potential of the novel polyelectrolytes derived from naphthalene diimide for achieving high-performance PSCs.

## 2. Results and Discussion

### 2.1. Synthesis of Materials and the Characterization

The synthesis route of materials and characterization details are described in the experimental section. The materials were characterized by ^1^H and ^13^C NMR and the spectra are shown in Supporting Information (Figures S1–S9). ^1^H NMR was internally referenced to the residual protonated solvent peak, and the ^13^C NMR was referenced to the central carbon peak of the solvent. In all spectra, chemical shifts are given in δ relative to the solvent. The polyelectrolytes were obtained by converting an amino side chain functionality within the naphthalene diimide unit into a quaternary ammonium salt. The neutral polymer was synthesized using a Suzuki coupling reaction between the hexylfluorene and naphthalene diimide unit. The neutral polymer had an average molecular weight of 31.7 kDa and a polydispersity index of 1.11.

**Figure 2:**
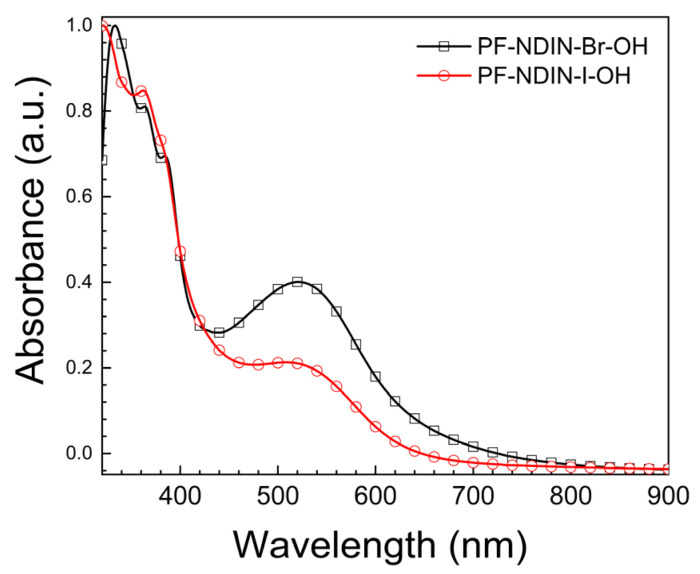
The spectrum of absorption in the UV-visible regions of polyelectrolyte films.

The optical properties of polyelectrolytes were measured through UV-Vis spectrophotometry (Figure 2). The spectra exhibited two wide absorption bands; the first absorption band at 350 to 400 nm represents to π–π* transition of the backbone, while the absorption band at 400 to 700 nm indicates the intramolecular charge transfer (ICT) between the hexylfluorene and naphthalene diimide unit [35,36]. The optical band gaps were calculated from the absorption edge and found to be 1.91 and 1.94 eV for PF-NDIN-Br-OH and PF-NDIN-I-OH, respectively. 

The determination of the HOMO and LUMO energy levels of PF-NDIN-Br-OH and PF-NDIN-I-OH was achieved by analyzing the onset oxidation and reduction potential observed in the cyclic voltammogram (Figure S10). Ag/AgNO_3_ was used as a reference electrode with a ferrocene/ferrocenium external standard. The HOMO and LUMO energies of PF-NDIN-Br-OH were calculated to be −5.42 and −3.72 eV, respectively. The HOMO and LUMO levels of PF-NDIN-I-OH were also found to be similar, with values of −5.44 and −3.72 eV, respectively. The relative HOMO and LUMO energies of the backbone are unaffected by the counteranion [37].

### 2.2. Investigation of the Surface Characteristics of ZnO/Polyelectrolyte

**Figure 3:**
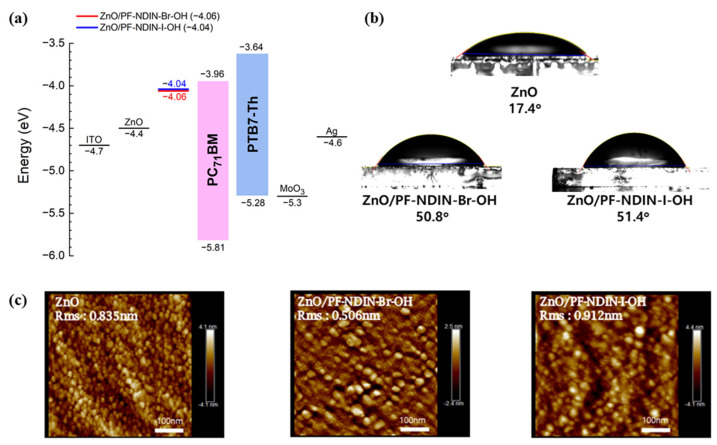
(**a**) The energy levels of the functional layers of the PSCs, (**b**) the water contact angle, and (**c**) AFM images of interlayer materials.

To assess the influence of the polymer on the work function (WF) of ZnO, an analysis with Kelvin probe microscopy was conducted (Figure 3a). The WF value measured for the device based on ZnO was −4.4 eV, while the ZnO/PF-NDIN-Br-OH and ZnO/PF-NDIN-I-OH exhibited higher WF values, which were −4.06 and −4.04 eV, respectively. Significantly, the relation between the shift in the WF of ZnO surfaces and the presence of the interfacial dipole was observed. Minimizing the energy offset at the interface is important because a specific energy offset can impede the efficient collection of charges. Consequently, the incorporation of a thin layer of polyelectrolytes will enhance the photovoltaic properties by decreasing the energy barrier at the interface and improving the collection of charge. A previous study indicates that the WF of the electrode decreases as the size of the CA increases [31]. With an increase in the size of the anion, there is a corresponding increase in the magnitude of the interfacial dipole and photovoltaic properties. The results illustrate that the CA size influences the interfacial dipole, resulting in a modified work function of the cathode. To examine the surface wettability of the ZnO modified by the polymer, a water contact angle (WCA) analysis was conducted (Figure 3b). The WCA of ZnO/PF-NDIN-Br-OH and ZnO/PF-NDIN-I-OH was shown to be 50.8° and 51.4°, respectively, which is higher than pristine ZnO (17.4°). This phenomenon arises due to the enhanced hydrophobicity resulting from the presence of the polymer layer on the ZnO surface. It implies that the polyelectrolytes have the potential to establish enhanced contact with the blend of the active layer. 

Atomic force microscopy (AFM) was used to evaluate the morphology of unmodified ZnO and ZnO coated with polyelectrolytes (Figure 3c). Apparently, ZnO/PF-NDIN-Br-OH demonstrated a uniform and smooth surface, exhibiting a lower root-mean-square (RMS) roughness of 0.506, compared to that of pristine ZnO (0.835 nm). A smaller root-mean-square (RMS) roughness value signifies a lower presence of pinholes in the film, suggesting a reduced number of traps in the interlayer. However, ZnO/PF-NDIN-I-OH demonstrated an RMS roughness of 0.912, which was the roughest surface among the interlayers. In general, a smooth and well-defined surface of the thin film is desirable for the subsequent deposition of the active layer.

### 2.3. Photovoltaic Properties

**Figure 4:**
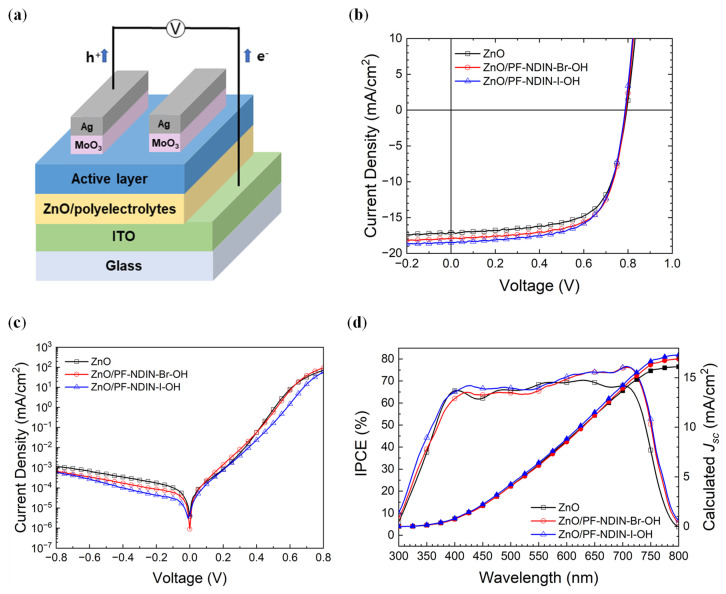
(**a**) The structure of inverted PSC, (**b**) J−V curves of PSCs under illumination, (**c**) under-dark conditions, (**d**) IPCE spectra with calculated $J_{sc}$.

The polyelectrolytes were employed as the cathode interlayer in an inverted PSC with the structure of ITO/ZnO/polyelectrolytes/PTB7-Th:PC_71_BM/MoO_3_/Ag (Figure 4a) to assess the photovoltaic properties. The PCE of an OSC is determined using three important parameters, namely $J_{sc}$, $V_{oc}$, and FF. Figure 4b,c show a common current–voltage (J−V) curve for an OSC under illumination and darkness. The photovoltaic properties are outlined in Table 1, providing an extensive overview of the device performance with different cathode interlayers. 

A PCE of 9.51% was obtained by utilizing PF-NDIN-Br-OH as the cathode interlayer, with a $J_{sc}$ of 17.9 mA cm^−2^, a $V_{oc}$ of 0.79 V, and an FF of 67.7%, leading to enhanced PCE compared to the ZnO-only-based device (8.96%). There are reports indicating that the presence of terminal hydroxyl groups in polyelectrolytes can enhance the interfacial properties of the device [30]. Incorporation of PF-NDIN-I-OH showed an improved PCE, up to 9.59%, compared to the device based on PF-NDIN-Br-OH due to the significant improvement in $J_{sc}$. The enhancement in $J_{sc}$ observed when employing polyelectrolytes as an interlayer aligns well with the corresponding WF value (Figure 3a). This also indicates a shift from a Schottky contact to an ohmic contact as the $J_{sc}$ increase [38]. It is important to note that the PCE exhibited a gradual increase with the enlargement of the CA size in polyelectrolytes [39]. A further analysis was conducted on the incident photon-to-current conversion efficiency (IPCE) spectra (Figure 4d). The calculated $J_{sc}$ of the devices based on ZnO/PF-NDIN-Br-OH and ZnO/PF-NDIN-I-OH was 16.1, 16.9, and 17.3 mA cm^−2^. The obtained $J_{sc}$ value aligns well with the values estimated from the IPCE spectra, thereby validating the accuracy of the device performances.

Electron-only devices were fabricated with the structure of ITO/ZnO/polyelectrolytes/PC_71_BM/LiF/Al to assess the electron mobilities ($\mu_{e})$ of the PSC. Employing the Mott–Gurney equation and the space–charge–limited current (SCLC) method, we calculated the $\mu_{e}$ of these devices [5]. Figure S11 depicts the characteristics of SCLC by illustrating the relationship between current density and electric field above the built-in voltage. The devices with PF-NDIN-Br-OH and PF-NDIN-I-OH exhibit the $\mu_{e}$ of 1.52 × 10^−3^ and 1.97 × 10^−3^ cm^−2^ V^−1^ s^−1^, respectively, which is higher than the $\mu_{e}$ of the ZnO-only device (8.83 × 10^−4^ cm^−2^ V^−1^ s^−1^). The enhanced $\mu_{e}$ is aligned well with the $J_{sc}$ value, which is indicative of the enhanced cathode modification capability [5].

The series resistance ($R_{s}$) was determined by evaluating the inverse slope of the J−V curve under 1.0 sun near the high current region. The devices based on PF-NDIN-Br-OH and PF-NDIN-I-OH exhibit smaller $R_{s}$ values of 1.37 Ω cm^2^ and 1.28 Ω cm^2^, respectively, compared to the ZnO-only-based device (2.01 Ω cm^2^). The presence of the polyelectrolyte layer results in a shift from a Schottky contact to an Ohmic contact at the cathode interfaces, which in turn leads to a reduction in the $R_{s}$ value. The device with PF-NDIN-Br-OH demonstrated higher shunt resistance ($R_{sh}$) values than the ZnO-only-based device (0.77 kΩ cm^2^), by minimizing short circuits between the contacts [30]. The devices incorporating PF-NDIN-I-OH demonstrate the lowest $R_{sh}$ values of 0.69 kΩ cm^2^. The values of the $R_{sh}$ align closely with the FF values. Lower FF values were observed when PF-NDIN-I-OH was applied in the device, suggesting a potential for leakage current in the device with PF-NDIN-I-OH as the interlayer [27]. Even though the device with PF-NDIN-I-OH has the lowest $R_{sh}$ values, the device exhibits the best $J_{sc}$, which mostly contributes to PCE enhancement.

**Figure 5:**
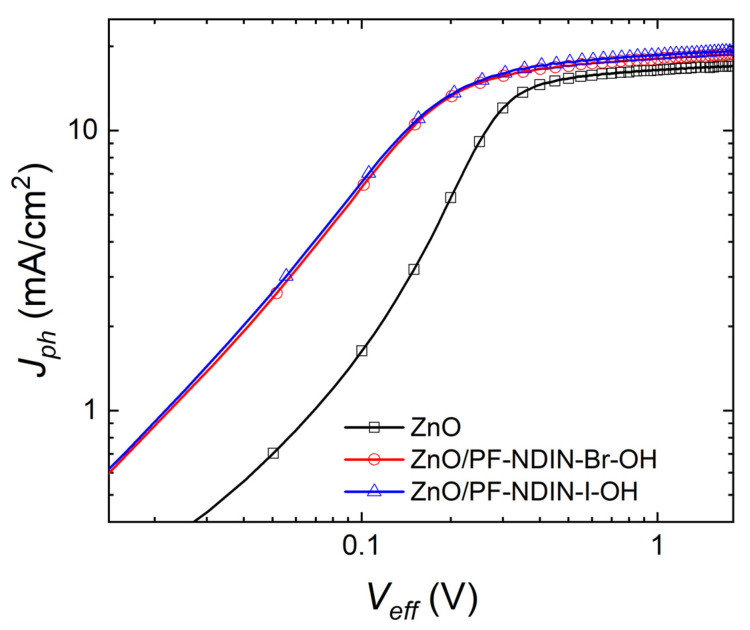
Photocurrent ($J_{ph}$) density on effective voltage ($V_{eff}$) with polyelectrolytes as interlayer.

The correlation of effective voltage ($V_{eff}$) with photocurrent density ($J_{ph}$) was analyzed to assess the mechanism of charge transport and collection in the devices. In Figure 5, a linear correlation is evident between the log ($J_{ph}$) and log ($V_{eff}$) with a low $V_{eff}$ range, which starts saturating at a high $V_{eff}$ region. The $V_{eff}$ values in the saturated photocurrent region ($V_{sat}$) based on PF-NDIN-Br-OH and PF-NDIN-I-OH were 0.21 and 0.20 V, respectively, which presented a decreased value compared to the pristine ZnO-based device (0.32 V). The result is aligned with the trend observed in the $J_{sc}$ and PCE. A lower $V_{sat}$ value implies a faster movement from the space–charge–limited current to the saturated region.

At higher values of $V_{eff}$, certain factors including the maximum exciton generation rate ($G_{max}$), exciton dissociation probability, charge mobility, and charge collection probability are correlated with the saturation current density ($J_{sat}$). The $G_{max}$ value equals to $J_{ph}$/q⋅L, where L represents the active layer thickness and q represents the elementary charge. The $G_{max}$ value of the ZnO was 1.58 × 10^28^ m^−3^ s^−1^ and the $G_{max}$ values exhibited from the device based on PF-NDIN-Br-OH and PF-NDIN-I-OH under the $J_{sat}$ condition were 1.66 × 10^28^ and 1.72 × 10^28^ m^−3^ s^−1^, respectively. The results suggest that there is a correlation between the observed trend in $J_{sc}$ and $G_{max}$. Furthermore, the exciton dissociation probability was determined using the ratio of $J_{ph}/J_{sat}$ at any $V_{eff}$ and found to be 85.0%, 88.5%, and 89.0% for the devices utilizing ZnO, PF-NDIN-Br-OH, and PF-NDIN-I-OH, respectively. This suggests charge recombination is lowered and enhances the charge collection at the cathode interface.

**Figure 6:**
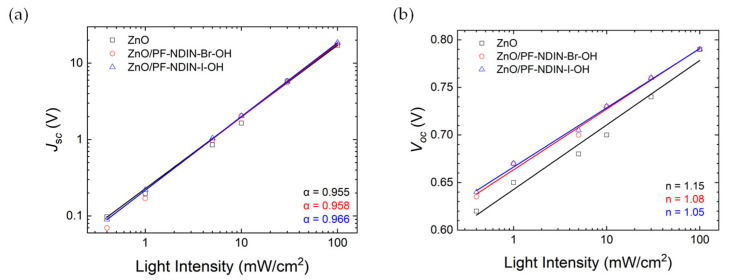
(**a**) The plots of $J_{sc}$ on light intensity and (**b**) $V_{oc}$ on light intensity with different interlayers.

The dependence of $J_{sc}$ and $V_{oc}$ on light intensity ($P_{light}$) was measured through $J_{sc}$ vs. $P_{light}$ and the $V_{oc}$ vs. $P_{light}$ curves to examine charge carrier recombination (Figure 6a). The equation $J_{sc}\propto {\left(P_{light}\right)}^{\alpha }$ can explain the correlation between $J_{sc}$ and $P_{light}$, where the α value tends to approach 1 when there is negligible bimolecular charge recombination [40]. The α values of the devices based on pristine ZnO, PF-NDIN-Br-OH, and PF-NDIN-I-OH were 0.955, 0.958, and 0.966, respectively. Typically, polymer solar cells demonstrate a power law dependence of $J_{sc}$ on light intensity in the range of 0.85 to 1. Deviation from α = 1 is often attributed to bimolecular recombination, leading to a slight loss of carriers [41,42].

Comprehending the recombination process is vital for enhancing $J_{sc}$ and thus improving the overall performance of PSCs, as recombination depletes photogenerated charge carriers. The Shockley–Read–Hall mechanism, also referred to as trap-assisted recombination, is a recombination process that arises when trap states emerge within the bandgap [42]. The slope of the $V_{oc}$ vs. $P_{light}$ curve shows the presence of trap-assisted recombination, and this is depicted in Figure 6b [43]. The relationship between $V_{oc}$ and $P_{light}$ is used to calculate n (ideality factor), which is explained by Voc∝nkBTqlnPlight , where $k_{B}$ is the Boltzmann constant, T is the temperature, and q is the elementary charge [43]. A dominance of band-to-band recombination corresponds to n=1, while an n value of 2 indicates the occurrence of the Shockley–Read–Hall recombination. The device utilizing pristine ZnO demonstrated an n value of 1.15, while the devices employing PF-NDIN-Br-OH and PF-NDIN-I-OH exhibited n values of 1.08 and 1.05, respectively. This suggests that polymers efficiently minimize the presence of traps. Additionally, it is crucial to analyze the n values as they follow a similar trend to the PCE value [5].

**Figure 7:**
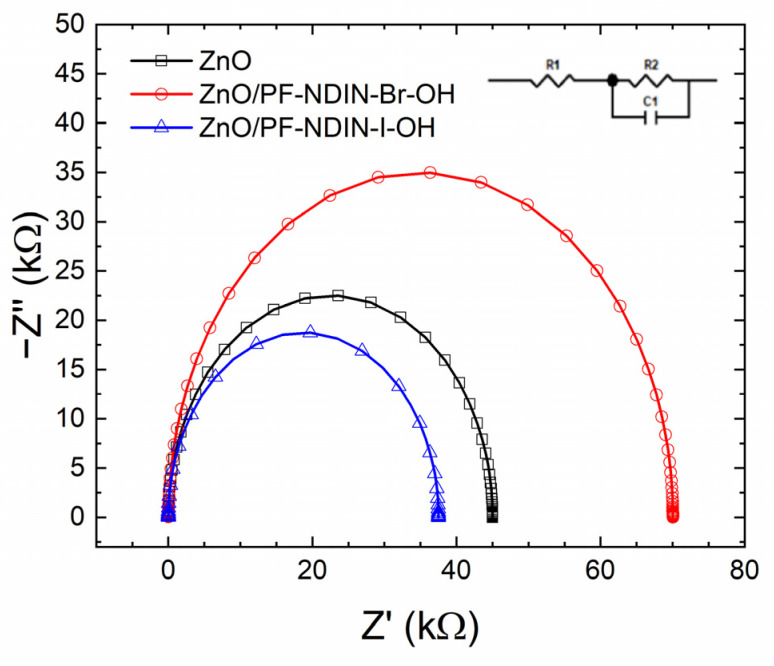
EIS spectra of PSCs.

To investigate the recombination process that occurs in the device, electrochemical impedance spectroscopy (EIS) was conducted. As depicted in Figure 7, the EIS spectra for significant recombination align with the Gerischer impedance model where the absence of a transmission line in the semi-circle suggests that large recombination has occurred in the devices. The recombination resistance ($R_{rec}$) was determined by fitting the data and the $R_{rec}$ rises proportionally with the size of the EIS semi-circle, signifying the accumulation of charges at the ZnO interface.

The $R_{rec}$ values at zero bias of the devices calculated at 0 V based on ZnO, PF-NDIN-Br-OH, and PF-NDIN-I-OH were 44, 70, and 37 kΩ, respectively. With increasing the semi-circle size, the $R_{rec}$ increased and aligned well with the FF of PSCs. Moreover, the EIS results establish a correlation with the $V_{OC}$ versus $P_{light}$ graph of the PSCs. The $R_{rec}$ is related to the recombination sites at the interface of the electrode/interlayer/active layer, with a higher $R_{rec}$ indicating a lower potential of carriers undergoing recombination.

## 3. Materials and Methods

### 3.1. Materials

Poly([2,6′-4,8-di(5-ethylhexylthienyl)benzo[1,2-b;3,3-b]dithiophene]{3-fluoro-2[(2-ethylhexyl)carbonyl] thieno[3,4-b]thiophenediyl}) (PTB7-Th) was purchased from Derthon Optoelectronic Material S&T CO., Ltd. (Shenzhen, China), and other chemicals were purchased from Sigma-Aldrich (St. Louis, MO, USA) or Fisher Scientific (Waltham, MA, USA) and were used as received unless otherwise described. (6,6)-Phenyl C71 butyric acid methyl ester (PC_71_BM, Cat No. nano-cPCBM-SF) was purchased from nano-C, Inc. (Westwood, MA, USA), respectively.

### 3.2. Synthesis of Compound ***1***

In a single-necked round-bottom flask, naphthalenetetracarboxylic dianhydride (2.68 g, 10 mmol) was slurried in concentrated sulfuric acid (25 mL) at ambient temperature, and the mixture was stirred at room temperature for 5 min to obtain a solution. 1,3-Dibromo-5,5-dimethylhydantoin (DBDMH) (3.57 g,15 mmol) was added in four portions over a period of 1 h at room temperature. The resulting brown solution was stirred at 50 °C for 10 h. The mixture was poured into crushed ice to precipitate the solid. The precipitated solid was filtered, washed with water and then with methanol, and finally dried under a vacuum to afford the crude product, which was further purified through crystallization from DMF. During crystallization, the partially ring-opened side product preferentially crystallized, leaving the supernatant with the product, which was directly used for the next step without purification. 

### 3.3. Synthesis of Compound ***2***

To a 50 mL 2-neck round-bottom flask fitted with a condenser, compound **1** (500 mg, 1.17 mmol) was added. The flask was pumped down to a vacuum and backfilled with N_2_ thrice. In total, 15 mL of glacial acetic acid was added. Dimethylaminopropylamine (299.8 mg, 2.93 mmol) was slowly added to the reaction mixture at room temperature. The reaction mixture was then heated to 130 °C under reflux for 30 min. Upon completion, the reaction mixture was quenched with ice, neutralized with Na_2_CO_3_, and extracted with CHCl_3_ (3 × 80 mL). The combined organic extracts were washed with deionized water (2 × 80 mL) followed by a brine solution (2 × 60 mL). A crude product was retrieved as yellow solids by evaporating the solvent. The crude product was further purified through recrystallization in ethanol to give pure 2. Yield: 279 mg, 40%. ^1^H NMR (400 MHz, CDCl_3_) δ (ppm): 8.98 (s, 2H), 4.26 (t, 4H), 2.45 (t, 4H), 2.24 (s, 12H), 1.92 (m, 4H). ^13^C NMR (400 MHz, CDCl_3_) δ (ppm): 162.9, 160.8, 139.1, 131.1, 128.4, 125.3, 124.0, 57.1, 45.3, 40.1, 25.8. Anal. Calcd for C_24_H_26_Br_2_N_4_O_4_ (%): C, 48.50; H, 4.41; Br, 26.89; N, 9.43; O, 10.77. Found: C, 48.76; H, 4.52; N, 9.60.

### 3.4. Synthesis of Compound ***3***

2,7-dibromofluorene (0.21 g, 0.648 mmol), 50wt% NaOH (5 mL), and a catalytic amount of tetrabutylammonium bromide (10 mol %) in 25 mL of DMSO were added to a flask under nitrogen. 1-Bromohexane (0.74 g, 4.536 mmol) was added and the mixture was heated at 60 °C continuously for 5 h. The reaction mixture was cooled to room temperature and extracted with chloroform. The organic layer was washed with water and dried over anhydrous magnesium sulfate. The solvent was removed under a vacuum, and the crude product was purified via column chromatography on silica gel using a hexane/dichloromethane solvent system to obtain the white solid. Yield: 0.31 g, 99%. ^1^H NMR (400 MHz, CDCl_3_) δ (ppm): 7.44–7.54 (m, 6H), 1.90–1.95 (m, 4H), 1.04–1.27 (m, 12H), 0.76–0.81 (t, 6H), 0.55–0.70 (m, 4H) ppm. ^13^C NMR (400 MHz, CDCl_3_) δ (ppm): 152.6, 139.1, 130.2, 126.3, 121.6, 121.1, 55.7, 40.3, 31.5, 29.7, 23.7, 22.7, 14.1. Anal. Calcd for C_25_H_32_Br_2_ (%): C, 60.99; H, 6.55; Br, 32.46. Found: C, 70.26; H, 6.72.

### 3.5. Synthesis of Compound ***4***

Anhydrous THF (50 mL) was added to compound **3** (2.00 g, 4.31 mmol) under an argon atmosphere and the mixture was cooled down to −78 °C. A 2.5 M solution of n-BuLi (5.17 mL, 12.9 mmol) was added dropwise and the solution was stirred at −78 °C for 1 h. 2-Isopropoxy-4,4,5,5-tetramethyl-1,3,2-dioxaborolane (4.23 mL, 20.7 mmol) was added and the solution was stirred for 15 min. The mixture was allowed to warm up slowly to room temperature and stirred overnight. Diethyl ether (150 mL) was added and the organic layer was washed with water (3 × 90 mL) and brine (90 mL), dried over magnesium sulfate, filtered, and concentrated under reduced pressure. The crude product was purified through flash chromatography on silica gel with hexane/ethyl acetate as the eluent to obtain the product as a colorless solid. Yield: 2.12 g, 88%. ^1^H NMR (400 MHz, CDCl_3_) δ (ppm): 7.80 (d, 2H), 7.75 (s, 2H), 7.71 (d, 2H), 2.13–1.84 (m, 4H), 1.39 (s, 24H), 1.15–0.89 (m, 12H), 0.74 (t, 6H), 0.63–0.46 (m, 4H); ^13^C NMR (400 MHz, CDCl_3_) δ (ppm): 150.63, 144.07, 133.79, 129.09, 119.51, 83.86, 55.33, 40.23, 31.58, 29.77, 25.09, 23.73, 22.70, 14.14. Anal. Calcd for C_37_H_56_B_2_O_4_ (%): C, 75.78; H, 9.63; B, 3.69; O, 10.91. Found: C, 75.86; H, 10.01.

### 3.6. Synthesis of PF-NDIN

In a 25 mL dry Schlenk tube, compound **2** (0.119 g, 0.2 mmol), compound **4** (0.117 g, 0.2 mmol), tetrakis(triphenylphosphine) palladium [(PPh_3_)_4_Pd(0)] (0.023 g), and one drop of Aliquat 336 were dissolved in a mixture of 3 mL of degassed toluene and 3 mL of a TEAOH solution (20%) under the protection of nitrogen. The mixture was stirred for 2 days at 95 °C under a nitrogen atmosphere. After the reaction mixture was cooled to room temperature, it was poured into 100 mL of methanol. The precipitated material was recovered via filtration through a funnel. The resulting solid material was washed with acetone to obtain a purple solid. Yield: 0.11 g, 80%. ^1^H NMR (400 MHz, CDCl_3_) δ (ppm): 7.85–7.32 (br, 2H), 7.17–6.74 (br, 6H), 3.87 (br, 4H), 1.85 (br, 8H), 1.35–0.88 (br, 16H), 0.88–0.43 (br, 22H).

### 3.7. The General Synthesis Procedure of PF-NDIN-Br-OH and PF-NDIN-I-OH

In total, 100 mg of PF-NDIN was dissolved in 3 mL of toluene, and excess bromoethanol or iodoethanol was added and then the mixture was stirred at 90 °C for 72 h. Then, the solution was concentrated and precipitated into a mixture of ethyl acetate. The purple solid was collected and dried. The isolated polyelectrolytes were obtained in 70–75% yields. 

^1^H NMR (400 MHz, MeOD) δ (ppm): 

PF-NDIN-Br-OH: 8.91–6.76 (m, 8H), 4.26 (br, 4H), 3.26–2.61 (br, 24H), 2.70–1.87 (br, 8H), 1.68–0.98 (br, 16H), 0.77 (br, 6H).

^1^H NMR (400 MHz, DMSO-d6) δ (ppm): 

PF-NDIN-I-OH: 8.93–6.78 (m, 8H), 4.26 (br, 4H), 3.35–2.71 (br, 24H), 2.76–1.90 (br, 8H), 1.70–1.06 (br, 16H), 0.80 (br, 6H).

### 3.8. Fabrication of OSCs

To fabricate the inverted-type OSC with the structure, the following applies: [ITO/ZnO (25 nm)/interlayer/active layer (PTB7-Th:PC_71_BM, 80 nm)/MoO_3_ (3 nm)/Ag (100 nm)]. The devices were prepared on indium tin oxide (ITO) (~150 nm)-coated glass substrates with 15 Ω/square of sheet resistance. ITO-coated glass substrates were cleaned through sonication in soap water, two-time deionized water, methanol, acetone, and isopropanol for 20 min each. Zinc acetate dihydrate (0.1 g) and 0.025 mL of ethanolamine were dissolved in 1 mL of methoxyethanol and stirred for 12 h at 60 °C. A thin film of a ZnO sol–gel precursor was spin-coated at 4000 rpm for 60 s and then annealed at 200 °C for 10 min. The interlayer solution was spin-coated at 2000 rpm for 60 s. The interlayer solution was filtered through a 0.45 um cellulose acetate membrane filter before spin coating. The active layer was spin-coated in the glove box at 1800 rpm for 60 s from a mixture of PTB7-Th and PC_71_BM obtained by dissolving 10 mg of PTB7-Th and 15 mg of PC_71_BM in 1 mL of chlorobenzene with 3% (*v*/*v*) 1,8-diiodooctane (DIO). The active solution was filtered through a 0.45 um PTFE membrane filter before spin coating. Successive layers of MoO_3_ and Ag were thermally evaporated through a shadow mask, with a device area of 0.09 cm^2^ at 2 × 10^−6^ Torr.

### 3.9. Fabrication of Electron-only Devices 

Electron-only devices with the structure [ITO/ZnO (25 nm) with or without polyelectrolytes (5 nm)/PC_71_BM (60 nm)/LiF (1 nm)/Al (100 nm)] have been fabricated to investigate the electron mobility with an interlayer or pristine ZnO layer.

### 3.10. Measurement

The ^1^H and ^13^C NMR spectra were measured on a JEOL JNM ECZ-400 spectrometer (JEOL Korea Ltd., Seoul, Korea). The elemental analysis of the synthesized compound was carried out on an Elementar Vario macro/micro elemental analyzer. UV–visible spectra of solutions and thin films were determined using a JASCO V-730. Gel permeation chromatography (GPC) was measured on an Agilent 1200 series (Agilent Technologies Korea, Ltd., Seoul, Korea) instrument with THF as the eluent. Cyclic voltammetry (CV) measurements were carried out by using a potentiostat (IVIUM Technology, COMPACTSTAT, HS Technologies, Gunpo, Korea) with tetrabutylammonium hexafluorophosphate (0.1 M, Bu_4_NPF_6_) as the electrolyte in dichloromethane. For CV measurements, a glassy carbon electrode coated with polyelectrolytes and a platinum wire was used as the working and counter electrode, respectively. Ag/AgNO_3_ was used as a reference electrode with a ferrocene/ferrocenium external standard. Non-modulated impedance spectroscopy was performed using an impedance analyzer (IVIUM Technology, Impedance Monitor, HS Technologies, Gunpo, Korea) at an applied bias. A 50 mV voltage perturbation was applied over a constant applied bias, in the frequency range between 1 Hz and 1.0 MHz under the dark condition with the device for current density–voltage (J-V) characteristics. The recombination resistances at an applied bias were deduced from equivalent circuit fitting. The thickness of the films was measured with an AlphaStep IQ surface profiler (KLA-Tencor Inc., Milpitas, CA, USA). The water contact angles of the substrates were measured using SEO Model Phoenix 300 (SEO, Suwon, Korea). Kelvin probe microscopy (KPM) measurements (KP technology Ltd., Model KP020, Wick, Scotland) were performed to measure the work function of ZnO layers with and without an interlayer, and the work function of the samples was estimated by measuring the contact potential difference between the sample and the KPM tip. The KPM tip was calibrated against a standard reference gold surface, with a work function of 5.1 eV. The morphology of the films was analyzed with Icon-PT-PLUS (BRUKER Co. Ltd., Billerica, MA, USA). The current density–voltage measurements were performed under simulated light (AM 1.5 G, 1.0 sun condition/100 mW/cm^2^) from a 150 W Xe lamp, using a KEITHLEY Model 2400 (Keithley Instruments, Solon, Ohio, USA) source-measure unit. A calibrated Si reference cell with a KG5 filter certified by the National Institute of Advanced Industrial Science and Technology was used to confirm the 1.0 sun condition. 

## 4. Conclusions

In conclusion, we have demonstrated the influence of the synergistic effects of polyelectrolytes with hydroxyl groups at the side chain and larger anions (e.g., iodide) when applied to PSCs. The device incorporating PF-NDIN-Br-OH demonstrated a PCE of 9.51%, while the device with PF-NDIN-I-OH achieved a slightly higher PCE of 9.59%. In contrast, the device based on ZnO-only as a reference showed a PCE of 8.96%. The presence of the ionic moiety at the side chain in this material leads to the formation of a beneficial interfacial dipole at the cathode interface, consequently improving the PCE compared to pristine ZnO. Due to the enhanced interfacial dipole introduced by the interlayer, a transition from a Schottky contact to an ohmic contact was observed, and this transition is closely associated with the shift in the WF of the ZnO. The PCE increased with the enlargement of the CA size in polyelectrolytes, which mainly resulted from the $J_{sc}$ enhancement. As PF-NDIN-I-OH yielded the highest PCE as the cathode interlayer, this outcome indicates a connection between device performance and the presence of a larger counteranion. This study demonstrated the potential of the novel polyelectrolytes derived from naphthalene diimide for achieving high-performance PSCs.

## Figures and Tables

**Table 1:** Photovoltaic properties of the devices with different cathode interlayers. The values given within parentheses represent the average values obtained from a set of measurements, with 10 devices being averaged for each value.

Interlayer	$J_{sc}$ (mA/cm^2^)	$V_{oc}$ (V)	FF (%)	PCE (%)	$R_{s}$ (Ω cm^2^)	$R_{sh}$ (kΩ cm^2^)
ZnO	17.1 (16.9 ± 0.4)	0.79 (0.79 ± 0.00)	66.3 (65.9 ± 0.6)	8.96 (8.77 ± 0.27)	2.01	0.77
ZnO/PF-NDIN-Br-OH	17.9 (17.8 ± 0.4)	0.79 (0.79 ± 0.00)	67.7 (67.7 ± 0.8)	9.51 (9.43 ± 0.15)	1.37	1.58
ZnO/PF-NDIN-I-OH	18.5 (18.4 ± 0.1)	0.79 (0.79 ± 0.00)	65.7 (65.7 ± 0.3)	9.59 (9.55 ± 0.03)	1.28	0.69

## Data Availability

The data are available on request from the corresponding author.

## Supplementary Materials

The following supporting information can be downloaded at: https://www.mdpi.com/article/10.3390/ijms25010522/s1.
