# Supplementary material for: Novel Polyelectrolytes Based on Naphthalene Diimide with Different Counteranions for Cathode Interlayers in Polymer Solar Cells
Source: Int J Mol Sci. 2023 Dec 30;25(1):522. doi: 10.3390/ijms25010522 (PMC10779300; doi:10.3390/ijms25010522)
Supplement: Supplementary file 1 [file ijms-25-00522-s001.zip › ijms-2781339-supplementary.pdf]

Supporting Information

Novel Polyelectrolytes Based on Naphthalene Diimide with Different Counteranions for Cathode Interlayers in Polymer Solar Cells

Rahmatia Fitri Binti Nasrun [1,2,†], Dong Hwan Son [1,2,†] and Joo Hyun Kim [1,2,*]

1 Department of Polymer Engineering, Pukyong National University, Busan 48513, Republic of Korea;
rahmatiaf@pukyong.ac.kr (R.F.B.N.); ehdghks507@naver.com (D.H.S.)
2 CECS Research Institute, Core Research Institute, Busan 48513, Republic of Korea.
* Correspondence: jkim@pknu.ac.kr
† These authors contributed equally to this work.

Keywords: polyelectrolytes; cathode interlayer; naphthalene diimide; polymer solar cell

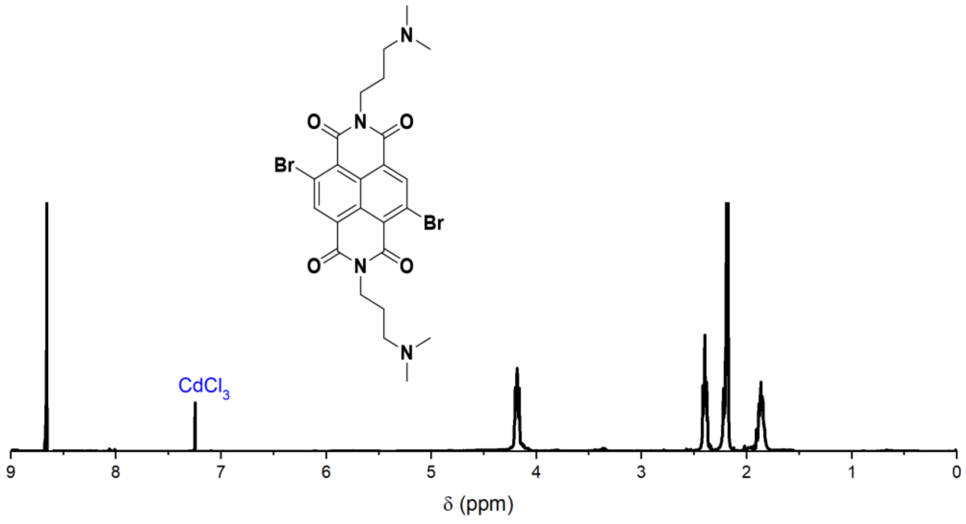

Figure S1. ^{1}H spectra of compound 2.

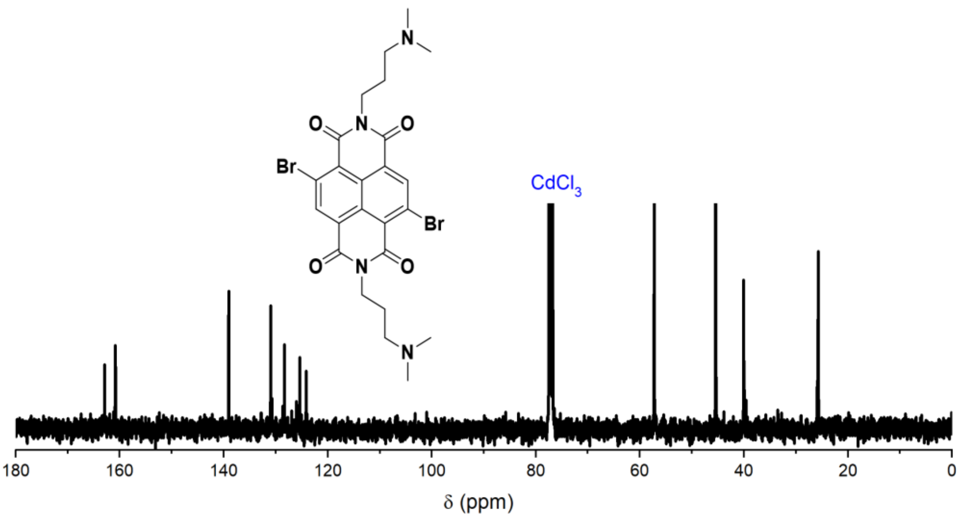

Figure S2. ^{13}C spectra of compound 2.

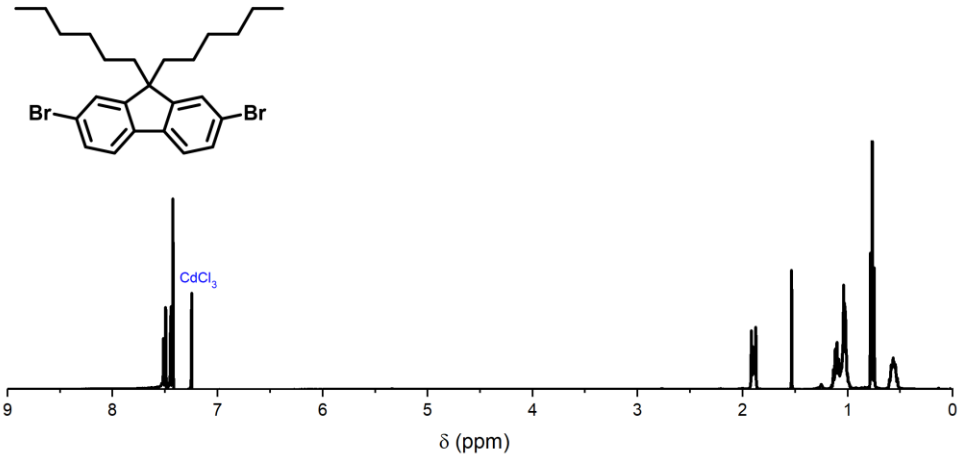

Figure S3. ^{1}H spectra of compound 3.

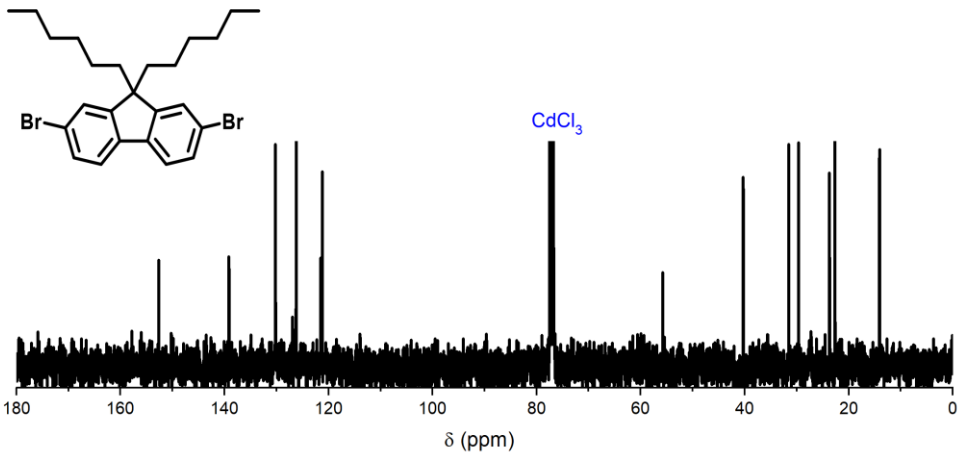

Figure S4. ^{13}C spectra of compound 3.

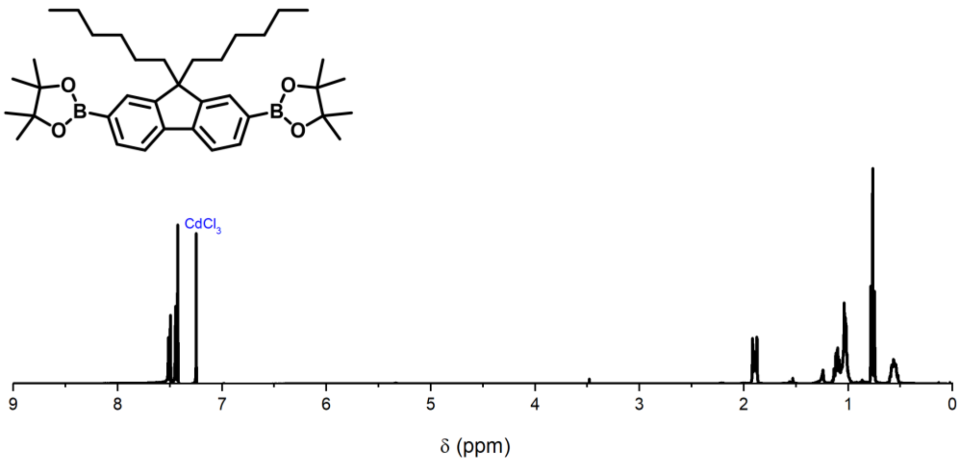

Figure S5. ^{1}H spectra of compound 4.

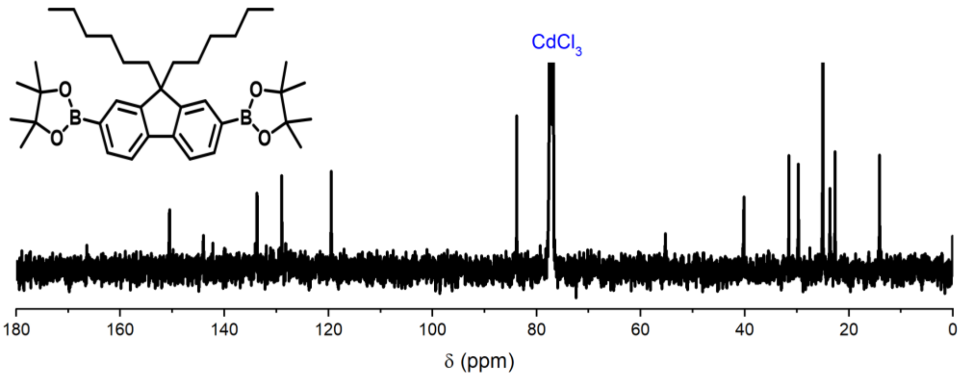

Figure S6. ^{13}C spectra of compound 4.

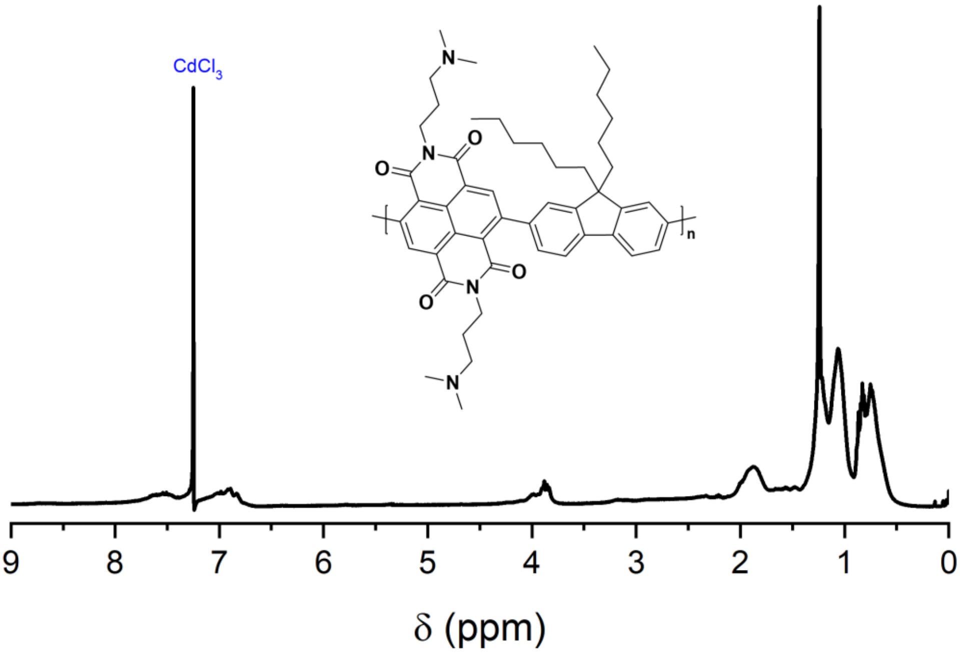

Figure S7. ^{1}H spectra of PF-NDIN.

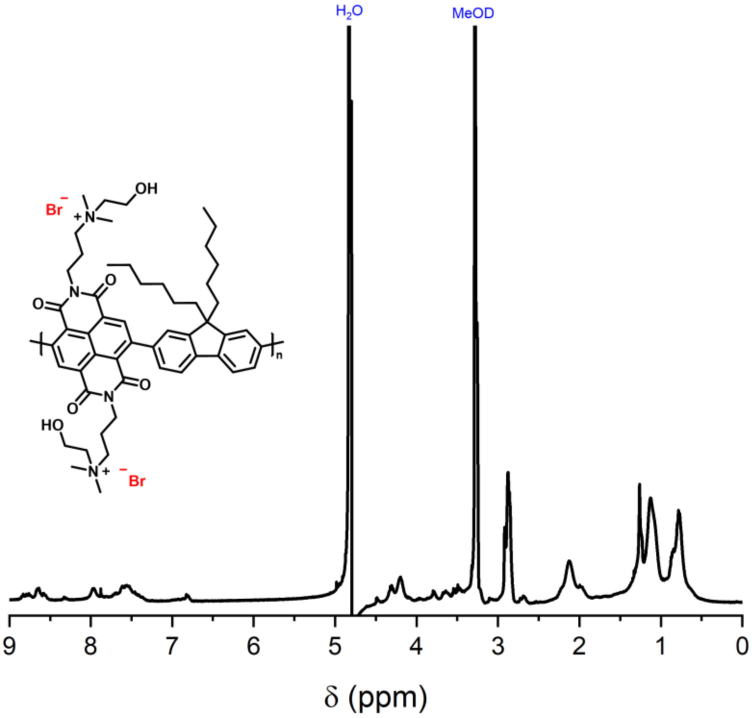

Figure S8. ^{1}H spectra of PF-NDIN-Br-OH.

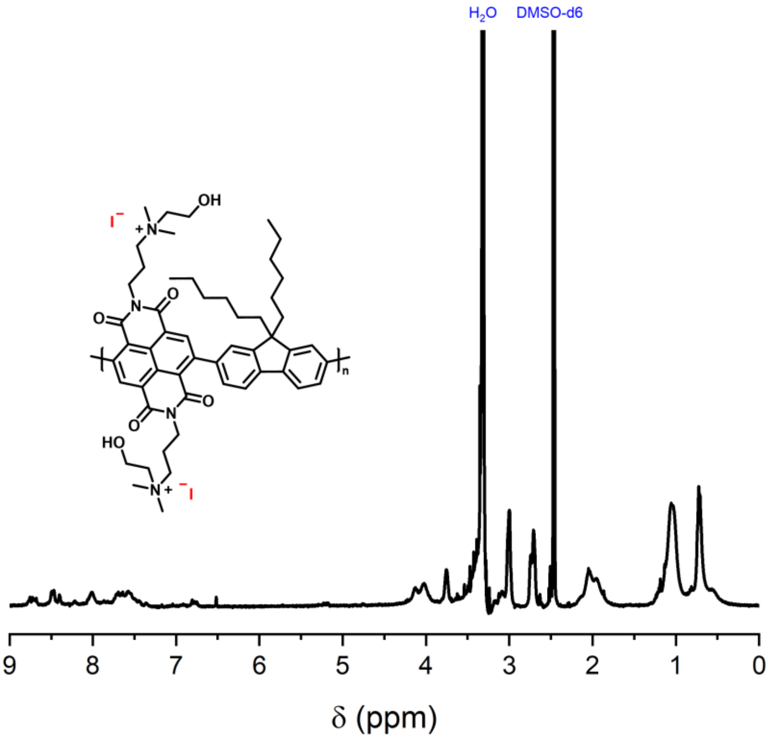

Figure S9. ^{1}H spectra of PF-NDIN-I-OH.

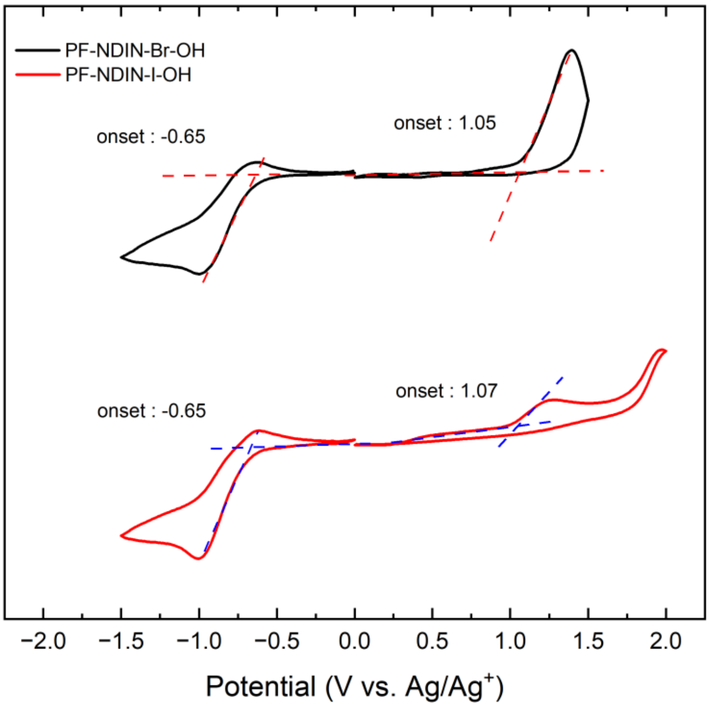

Figure S10. Cyclic voltammogram of polyelectrolytes.

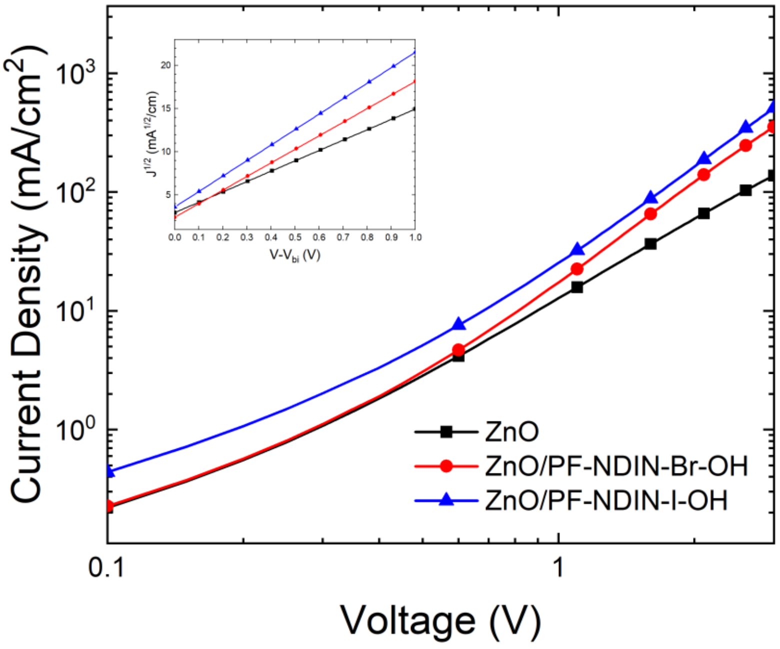

Figure S11. Current density–voltage curves of the electron-only devices with fitted line (V, applied voltage; V_{bi}, built-in voltage).

Table S1. The list of abbreviations in the paper

Word	Abbreviations
Polymer solar cells	PSCs
Power conversion efficiency	PCE
Short-circuit current	J_{SC}
Open-circuit voltage	V_{OC}
Fill factor	FF
Lowest unoccupied molecular orbital	LUMO
Highest occupied molecular orbital	HOMO
Poly([2,6′-4,8-di(5-ethylhexylthienyl)benzo[1,2-b;3,3-b]dithiophene]{3-fluoro-2[(2-ethylhexyl)carbonyl]thieno[3,4-b]thiophenediyl})	PTB7-Th
(6,6)-Phenyl C71 butyric acid methyl ester	$PC_{71}BM$
Ultraviolet–visible	UV–Vis
Intramolecular charge transfer	ICT
Cyclic voltammogram	CV
Work function	WF
Incident photon-to-current efficiency	IPCE
Series resistance	R_s
Shunt resistance	R_{sh}
Photocurrent density	J_{ph}
Effective voltage	V_{eff}
Saturated photocurrent regime	V_{sat}
Saturation current density	J_{sat}
Maximum exciton generation rate	G_{max}

Electron charge	q
Thickness of the active layer	L
Electron mobility	μ_e
Light intensity	P_{light}
Electrochemical impedance spectroscopy	EIS
Recombination resistance	R_{rec}
